# Preparation and Biological Study of ^68^Ga-DOTA-alendronate

**DOI:** 10.7508/aojnmb.2016.02.006

**Published:** 2016

**Authors:** Ashraf Fakhari, Amir R. Jalilian, Fariba Johari-Daha, Mehdi Shafiee-Ardestani, Ali Khalaj

**Affiliations:** 1 Tehran University of Medical Sciences, Faculty of Pharmacy, Tehran, Iran; 2 Nuclear Science and Technology Research Institute (NSTRI), Tehran, Iran

**Keywords:** Alendronate, Biodistribution, DOTA, Ga-68, Radiolabeling

## Abstract

**Objective(s):**

In line with previous research on the development of conjugated bisphosphonate ligands as new bone-avid agents, in this study, DOTA-conjugated alendronate (DOTA-ALN) was synthesized and evaluated after labeling with gallium-68 (^68^Ga).

**Methods:**

DOTA-ALN was synthesized and characterized, followed by ^68^Ga-DOTA-ALN preparation, using DOTA-ALN and ^68^GaCl_3_ (pH: 4-5) at 92-95° C for 10 min. Stability tests, hydroxyapatite assay, partition coefficient calculation, biodistribution studies, and imaging were performed on the developed agent in normal rats.

**Results:**

The complex was prepared with high radiochemical purity (>99% as depicted by radio thin-layer chromatography; specific activity: 310-320 GBq/mmol) after solid phase purification and was stabilized for up to 90 min with a log P value of -2.91. Maximum ligand binding (65%) was observed in the presence of 50 mg of hydroxyapatite; a major portion of the activity was excreted through the kidneys. With the exception of excretory organs, gastrointestinal tract organs, including the liver, intestine, and colon, showed significant uptake; however, the bone uptake was low (<1%) at 30 min after the injection. The data were also confirmed by sequential imaging at 30-90 min following the intravenous injection.

**Conclusion:**

The high solubility and anionic properties of the complex led to major renal excretion and low hydroxyapatite uptake; therefore, the complex failed to demonstrate bone imaging behaviors.

## Introduction

High morbidity and mortality rates of malignant diseases highlight the necessity of prompt diagnostic modalities, including positron emission tomography (PET). Limited access to radionuclide sources, such as cyclotrons and reactors, has led to the development of generator-based PET radiopharmaceuticals.

In general, the high cost of installing and running cyclotrons in nuclear medicine centers is a source of major concern, especially in developing countries. Gallium-68 (^68^Ga) generator is recognized as the most widely applicable PET generator. In fact, many generators have been developed by different companies, based on the organic and inorganic solid phases (1-3).

Bone metastases are common in the progression of various malignancies, such as prostate, breast, and lung carcinoma, often entailing progressive pain (4). Bisphosphonates such as alendronic acid (ALN) used in clinics have shown significant inhibitory binding constants (Ki) on bone resorption enzymes.

The idea of developing bone-avid agents for bone metastasis imaging has been of great interest to researchers. ^68^Ga-based bone radiopharmaceuticals, including ^68^Ga-EDTMP (5, 6) and ^68^Ga-BPAMD (7), have demonstrated remarkable preclinical and clinical performance. On the other hand, various ongoing research studies are focusing on more efficient radiotracers with rapid bone uptake, rapid washout, and reduced unnecessary soft tissue uptake.

Accordingly, another interesting approach is to develop bisphosphonate ligands, including metal chelating agents, such as diethylenetriaminepentaacetic acid (DTPA) moiety, as previously reported for the ^99m^Tc-labeled analog (8). However, many DTPA-based ALN ligands show no bone uptake with rapid renal excretion, possibly due to the anionic properties of DTPA moiety and its high water solubility (9, 10).

**Figure 1:**
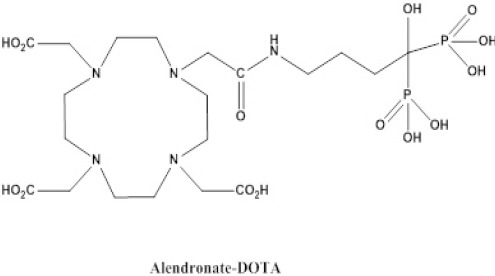
Chemical structure of DOTA-ALN

According to previous research, a polycyclic dentate, containing a bisphosphonate ligand (DOTA), could be considered as a possibly suitable chelate to develop new bone-avid agents. This chelate forms stable chelates with various metals, including lanthanides, copper, and gallium and is developed by the conjugation of DOTA-NHS and [4-amino-1-hydroxy-1-(hydroxy-oxido-phosphoryl)-butyl] phosphonic acid (ALN) (Figure 1).

In this study, we aimed to report the synthesis, characterization, and radiochemistry of [^68^Ga]-DOTA-ALN (Figure 1). For this purpose, biological evaluations and imaging studies were performed on normal rats.

## Methods

The ^68^Ge/^68^Ga generator (30 mCi/day) was obtained from Pars Isotope Co. (Karaj, Iran). Also, the chemicals were purchased from Aldrich Chemical Co. (Germany). Thin-layer chromatography (TLC) for the quality control of DOTA-ALN was performed on polymer-backed silica gel (TLC ready-to-use foil, F1500/LS 254, 20×20 cm, Schleicher & Schuell®, Germany).

ALN sodium was provided by Modava Company (Karaj, Iran). Normal saline and sodium acetate used for radiolabeling were of high purity and filtered through 0.22 µm ActiveX filters. Instant thin-layer chromatography (ITLC) was performed by counting Whatman No. 2 papers, using a thin-layer chromatography scanner (Bioscan AR2000, Bioscan Europe Ltd., France).

Biodistribution data were acquired by counting normal saline-washed tissues after weighing on a Canberra™ high-purity germanium (HPGe) detector (model GC1020-7500SL); radionuclidic purity was assessed with the same detector. For activity measurement of the samples, a Capintec CRC radiometer (NJ, USA) was used.

Animal studies were performed in accordance with the United Kingdom Biological Council’s Guidelines on the Use of Living Animals in Scientific Investigations (second edition). Images were acquired in the coincidence mode of a dual-head single-photon emission computed tomography (SPECT) system (DST-XL, Sopha Medical Vision, France).

### Synthesis of DOTA-ALN

DOTA-ALN was synthesized by the conjugation of DOTA-NHS ligand to ALN base at equimolar levels. Briefly, ALN in form of monosodium triphosphate (1 mmol) was dissolved in 0.5 ml of 0.1 M HCl for better solubility at 25°C, followed by the addition of a few drops of trimethylamine. Then, DOTA-NHS (1 mmol) was dissolved in 0.5 ml of deionized water and stirred at 35-40°C for 20 min. The ALN solution was added dropwise to the latter mixture over 20 min, and the final mixture was stirred at 35°C for 24 h.

The excess amount of ALN was extracted by ethanol. For more purification, the yellow solid was washed several times with ether, methanol, and ethanol (yield: 80%; m.p.: 165-168°C, IR: 1677 (-CO-N-), ^1^HNMR: δ: 3.12 (s, 6H, -CH_2_), 2.82 (s, 2H, -CH_2_), 1.98 (m, 2H, -CH_2_), 1.75 (m, 16H, -CH_2_), 1.27 (M, 4H, -CH_2_), MS: Calcd. 656, Obsd. 656 (M^++^H_2_O).

### ^68^Ge/^68^Ga generator and quality control

The detailed quality control and application of the generator have been recently reported (6). A prototype ^68^Ge/^68^Ga SnO_2_-based generator (30 mCi) was purchased from Pars Isotope Co. (Tehran, Iran). For radiolabeling, the generator was eluted with 0.6 M of HCl. The eluate was analyzed to control radionuclide purity, using gamma spectroscopy with an HPGe detector, coupled with a Canberra™ multi-channel analyzer for 1000 sec. The breakthrough was measured by counting the same sample for 48 h after determining the small amount of ^68^Ge in the sample.

Chemical purity control was carried out to determine the amounts of Sn, Zn, Fe, Ge, and Ga ions, using the inductively coupled plasma atomic emission spectroscopy (ICP-OES) method. Radiochemical purity was assessed by ITLC chromatograms of ^68^GaCl_3_ solution in 10% ammonium acetate:methanol mixture on silica gel sheets. Also, 10 mM of DTPA solution (pH~4) was used on a Whatman No. 2 paper to check the percentage of colloidal ^68^Ga fraction and/or non-cationic gallium species.

### Preparation of ^68^Ga-DOTA-ALN

In a typical run, the acidic solution, containing 0.3 ml of [^68^Ga]GaCl_3_ (197.95 MBq, 5.35 mCi), was transferred to a 5 ml borosilicate vial, containing 150 mg of solid HEPES, and stirred at room temperature for 1 min. Afterwards, 100 µl of DOTA-ALN stock solution in deionized water (4 mg/ml ≈ 0.63 *μ*mol) was added to the gallium-containing vial and vortexed at 90±C.

The active solution was assessed in terms of radiochemical purity by RTLC method at 5, 10, 20, and 30 min, respectively after labeling. The reaction mixture was then injected into a Sep-Pak C18 cartridge (Waters, USA), preconditioned with ethanol and water (2:5 ml), according to the manufacturer’s protocol. The column was then flushed with air (10 ml), and the amount of trapped and passed activity was measured. The column was finally eluted with 0.5 ml fractions of ethanol:normal saline (1:9) solution, and the fraction with maximum activity was passed through a 0.22 μ antimicrobial filter into a sterilized vial.

The pH of the active solution was 5.5-6. A 5 μl sample of the final solution was spotted on the silica gel paper, and 10% ammonium acetate:methanol (1:1) mixture was used as the mobile phase. The ascent of the desired compound on the paper was compared with RTLC of [^68^Ga]GaCl_3_ and [^68^Ga]-DOTA.

### Calculation of partition coefficient

The partition coefficient (logP) of ^68^Ga-DOTA-ALN was calculated, followed by the measurement of the specific activity ratio of organic and aqueous phases (P). A mixture of 1-octanol (1 ml) and isotonic acetate-buffered saline (1 ml, pH: 7), containing approximately 3.7 MBq of the radiolabeled gallium complex, was vortexed for 1 min at 37°C. Following centrifugation at > 1200 g for 5 min, octanol and aqueous phases were sampled and counted in an automatic well-type counter.

### In vitro stability of ^68^Ga-DOTA-ALN in the presence of human serum

The final solution (200 μCi, 50 μl) was incubated in the presence of freshly prepared human serum (300 μl) (Iranian Blood Transfusion Organization, Tehran, Iran) and maintained at 37°C for 2 hours. Every 30 min, 100 μl of 10% trichloroacetic acid was added to a portion of the mixture (50 μl), and the final mixture was centrifuged at 3000 rpm for 5 min, followed by decanting the supernatant from the debris. Stability was determined by performing frequent ITLC analysis of the supernatant, using the abovementioned ITLC system.

### Hydroxyapatite (HA) binding assay

The HA binding assay was performed according to the procedure described in the literature (11), with slight modifications. In brief, 2 ml of saline solution (pH: 7.4) was added to vials, containing 1.0, 2.0, 5.0, 10.0, 20.0, and 50.0 mg of solid HA, respectively, and the mixture was shaken for 1 h. Then, 50 microliter of the radioactive preparation was added, and the mixture was shaken for 1 h at room temperature.

The suspension was centrifuged, and two aliquots of the supernatant liquid were obtained from each vial; radioactivity was measured with a well-type counter. Experiments were performed using a similar procedure in the absence of HA. The binding percentage to HA was calculated, using the following formula:

HB=1-A/B×100

where A is the mean radioactivity of the supernatant sample under study, and B denotes the mean total value of whole activity.

### Biodistribution in wild-type rats

Distribution of the radiolabeled complex, as well as free ^68^Ga cations, was determined in rat tissues. The total amount of radioactivity injected in each rat was measured by counting the 1 ml syringe before and after injection in a dose calibrator with fixed geometry. The animals were sacrificed by CO2 asphyxiation at specific time intervals after injection (n=5 for each time interval).

The tissues (blood, heart, lung, brain, intestine, faeces, skin, stomach, kidneys, liver, muscle, and bone tissues) were weighed and rinsed with normal saline. Their specific activities were determined with a HPGe detector, equipped with a sample holder. The values were expressed as the percentage of the injected dose per gram of tissue.

### Imaging of ^68^Ga-DOTA-ALN in wild-type rats

The volume of the final ^68^Ga-DOTA-ALN solution (0.1 ml; 4.4 MBq activity) was injected into the dorsal tail vein of healthy rats. The total amount of the radioactive material, injected in each rat, was measured by counting the 1 ml syringe before and after injection, using an activity meter with fixed geometry.

The animals were relaxed by halothane and fixed in a suitable probe. Images were taken at 30, 60, and 90 min following the administration of the radiopharmaceutical in the coincidence mode. The useful field of view (UFOV) was 540×400 mm. The spatial resolution in the coincidence mode was 10 mm FWHM at CFOV, and sensitivity was 20 Kcps/µCi/cc. In total, 64 projections were acquired (30 s per view) with a 64×64 matrix.

## Results

### Production of the precursor

**Figure 2:**
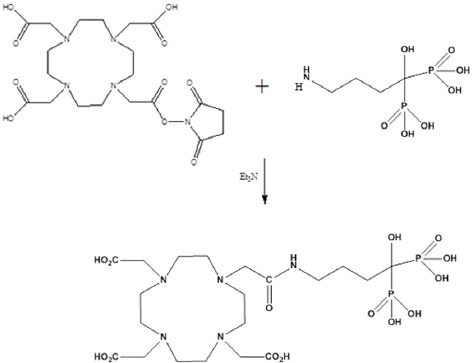
Reaction steps for the preparation of DOTA-ALN conjugate

Various reaction conditions were applied for DOTA-ALN conjugation. Conjugation was performed in dimethylformamide (DMF), dimethyl sulfoxide (DMSO), and aqueous mixtures. The most appropriate reaction solvent was shown to be water, since reaction work-up seemed feasible without precipitation/purification processes or any concern about residual solvents (Figure 2). For better yields, the ALN-free base was added to DOTA-NHS (not vice versa).

### Quality control of ^68^Ga elution

For the quality control of ^68^GaCl_3_ solution, a time-activity study was performed on the eluted sample after more than 10 half-lives of ^68^Ga in order to check ^68^Ge breakthrough. The data were recorded for eight days after elution. Calculations showed that the ^68^Ge/^68^Ga activity ratio was 1.6600×10^-5^ at the time of elution.

The concentrations of tin (from the generator material), iron (from the sealing parts and acid impurities), zinc (as a decay product), and gallium (as the target material) were determined, using the ICP-OES method, except for iron with a concentration of nearly 0.23 mg/L; the concentration of the rest of ions was lower than 0.1 mg/L. The radiochemical purity of ^68^GaCl_3_ solution was assessed in two solvents, as shown in Table 1.

**Table 1:** Chromatographic properties of various ^68^Ga species in the final elution

Species	Solvent 1	Solvent 2
^+68^Ga^3^	0.8	0.05
^-68^GaCl_x_	0.1	0.7
Colloids	0.05	0.05

### Radiolabeling

**Figure 3:**
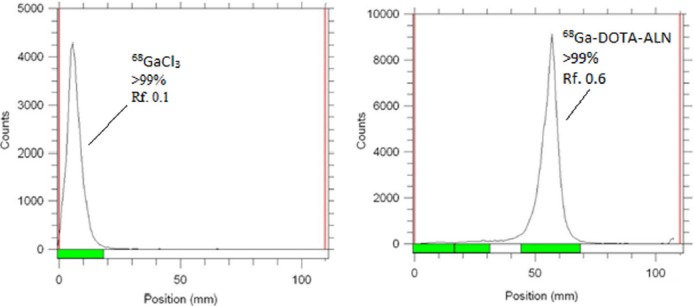
Radio thin-layer chromatography (RTLC) of ^68^Ga-GaCl_3_ (left) and ^68^Ga-DOTA-ALN (right) on silica gel papers developed in 10% ammonium acetate: methanol (1:1) mixture as the mobile phase under optimized conditions

A series of reactions were performed in order to reach the minimum required amount of the ligand; consequently, 0.5-0.6 µM of the ligand was applied for a typical labeling. Figure 3 demonstrates the chromatograms of ^68^Ga-DOTA-ALN complex, as well as free ^68^Ga under optimized conditions.

### Partition coefficient

As expected from chemical behaviors, the lipophilicity of ^68^Ga-DOTA-ALN was low due to the presence of three carboxylic groups in the DOTA moiety. The measured octanol-water partition coefficient (P) for the complex was found to depend on the pH of the solution. Based on the findings, at a pH of 7, the logP for the complex was -2.91.

### Stability

**Figure 4:**
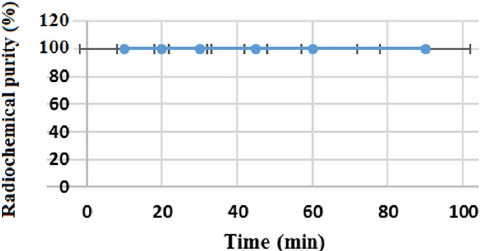
Radiochemical purity of the final complex in the final solution over 90 min

The chemical stability of ^68^Ga-DOTA-ALN was high enough to perform further analyses. Incubation of the complex in freshly prepared human serum (for 2 h at 37°C) showed no loss of ^68^Ga from the complex. The radiochemical purity of the complex remained at the initial level for 2 h under physiological conditions (Figure 4).

### HA assay measurements

**Figure 5:**
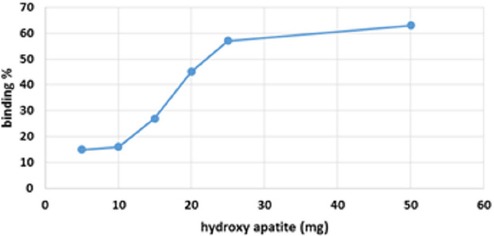
Binding of ^68^Ga-DOTA-ALN in the presence of 5-50 mg of HA

The HA assay demonstrated the low binding capacity of ^68^Ga-DOTA-ALN complex to HA. Even with 50 mg of HA, less than 70% binding was observed. The high solubility and anionic properties of the complex led to low HA binding; therefore, bone-avidity behaviors were not observed in vitro (Figure 5).

### Biodistribution

**Figure 6:**
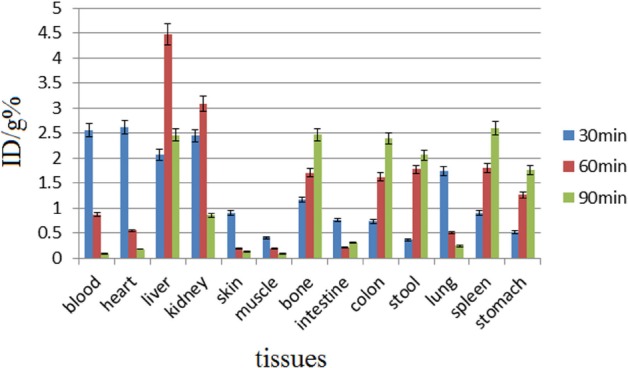
Biodistribution of [^68^Ga]GaCl_3_ (37 MBq, 100 µCi) in wild-type rats at 30-90 min following the intravenous injection through the tail vein (n=5; %ID/g: percentage of the injected dose per gram of tissue calculated based on the area under the curve of 511 keV peak in the gamma spectrum)

**Figure 7:**
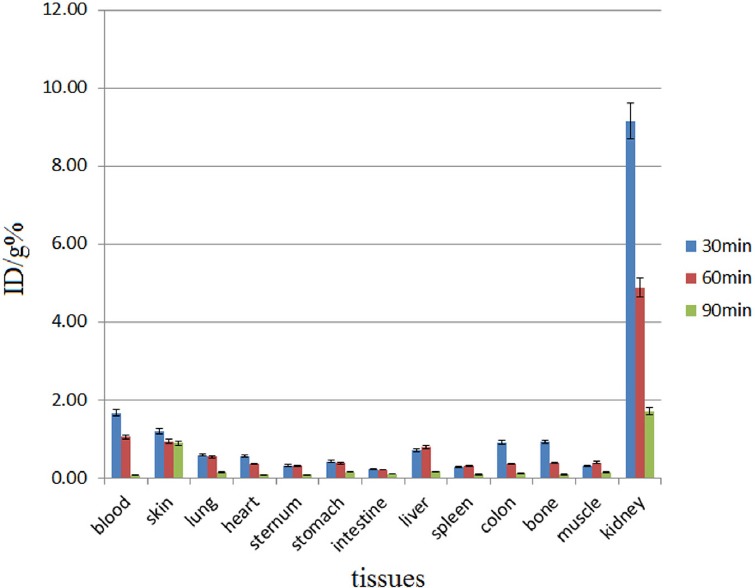
Biodistribution of ^68^Ga-DOTA-ALN (37 MBq, 100 µCi) in wild-type rats at 30-90 min following intravenous injection through the tail vein (n=5; %ID/g: percentage of the injected dose per gram of tissue calculated based on the area under the curve of 511 keV peak in the gamma spectrum)

**Figure 8:**
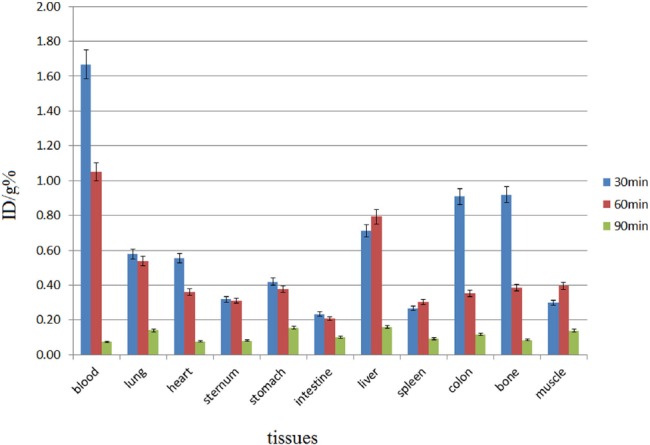
Biodistribution of ^68^Ga-DOTA-ALN (37 MBq, 100 µCi) in wild-type rats at 30-90 min following intravenous injection through the tail vein except for the kidneys (n=5; %ID/g: percentage of the injected dose per gram of tissue calculated based on the area under the curve of 511 keV peak in the gamma spectrum)

Biodistribution studies were performed on ^68^Ga-DOTA-ALN and free Ga^3+^ (Figure 6). The radiolabeled complex was rapidly washed from blood circulation into the urinary tract with a very low uptake in the liver (Figure 7). For a better understanding of the uptake of other organs (except for kidneys), Figure 8 is presented.

### Imaging studies

**Figure 9:**
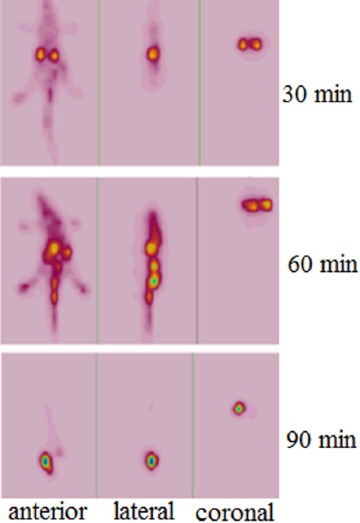
Static images of ^68^Ga-DOTA-ALN in normal rats at 30, 60, and 120 min after the injection (4.4 MBq for each rat)

^68^Ga-DOTA-ALN imaging in wild-type rats is presented in Figure 9. The results were comparable with tissue dissection studies.

## Discussion

The radiochemical purity of ^68^GaCl_3_ solution was assessed in two solvents. In 10 mmol/L of DTPA aqueous solution (solvent 1), free ^68^Ga^3+^ was coordinated to a more lipophilic moiety, as ^68^Ga(DTPA)^2-^ migrated to a higher Rf. The small radioactive fraction remaining at the origin could be attributed to colloids, since in the presence of very strong complexing agents (i.e., DTPA), ionic species other than ^68^Ga(DTPA)^2-^ can be rarely found.

Moreover, 10% ammonium acetate:methanol (1:1) mixture (solvent 2) was used for determining the radiochemical purity. The fast eluting species was possibly ^68^Ga, and other ionic forms of ^68^Ga, such as ^68^GaCl_4_^-^ (if existing), as well as colloids (not detected), remained at the origin (Rf= 0). The differences in impurity peaks in the two chromatograms could be partially related to colloidal impurity, which was insignificant. Also, the insignificant amount of activity (about < 1%) could be attributed to other ionic impurities.

Many considerations should be taken into account for radiolabeling. Some factors are known to influence the radiochemical purity of DOTA complex, while some change the quality of formulation for future applications (12). The elution portfolio of the generator is important, since more activity/eluted volume result in samples with higher specific activities for radiolabeling. On the other hand, use of 1 M or higher concentrations of HCl solutions usually yields a better radioactivity peak/elution, although other gallium species, which are not involved in radiolabeling, are formed at these concentrations.

In the presents study, by the selection of a suitable concentration (0.6-0.7 M), the major part of the daily-eluted generator activity was assessed; the peak of maximum activity ranged between 0.5 and 1.5 ml of the first elution. In the literature, the pH of radiolabeling for most ligands has been shown to be within the range of 4-5. Consequently, Ga activity eluted with 0.6-0.7 M of HCl usually exhibits a low pH (range: 1-2), which does not allow for the direct use of elution in the radiolabeling process. Therefore, addition of the calculated amount of solid HEPES to the elution has been proposed as a fast and reliable method.

The presence of metal impurities affects the complexation reaction due to impurities at ppm levels, compared to Ga-68 cations at ppb levels (or even lower levels). The radiolabeling yield usually decreases with the competition of other cations, particularly ferric cation in the radiolabeling reaction, leading to not only lower specific activities, but also a competition at the receptor level. Accordingly, the amount of cation release from the generator should be checked not only in the beginning of the generator application, but also in a routine manner in every center.

Long-term radiolysis reaction in the matrix of the generator is a major problem. Also, the interaction of Zn and Ga with DOTA moiety is reported to lead to an unwanted reaction with the conjugated peptide. In fact, routine (daily or twice a week) elution of the generator, even if not used for radiolabeling, would help to remove the unwanted cold cations present in the radiolabeling eluent.

In biodistribution studies, ^68^Ga is majorly excreted from the gastrointestinal tract with high blood contents due to transferrin binding at early time intervals. In the present study, significant colon, bone, and stomach activities were observed, except in kidneys which are not significant accumulation sites (Figure 6). The radiolabeled complex was rapidly washed from blood circulation into the urinary tract with a very low uptake in the liver (Figure 7). For a better understanding of the uptake of other organs except kidneys, the tracer biodistribution in other organs is presented in Figure 8 (after excluding the kidney uptake).

Based on the findings, less than 1% of the activity was accumulated in the bones. ^68^Ga-DOTA-ALN imaging in wild-type rats showed highly comparable results with tissue dissection studies at 0.5 h following the injection, and the findings indicated high kidney uptake. However, at 1 h following the injection, kidney and bladder uptake was observed, although most of the activity was at the baseline level within 120 min (mostly detected in the bladder only) (Figure 8).

^68^Ga-DOTA-ALN imaging in wild-type rats is presented in Figure 9. The results were comparable with tissue dissection studies. At 0.5 h after the injection, high kidney uptake was observed, while after 1 h following the injection, kidney and bladder uptake was reported, although most of the activity was at a baseline level after 120 min (mostly detected in the bladder only).

## Conclusion

DOTA-ALN was completely synthesized and characterized in the present study. ^68^Ga-DOTA-ALN was prepared within 7-10 min at 95°C with more than 99% radiochemical purity and 310-320 GBq/mmol specific activity. Also, water solubility properties and acceptable stability after solid phase purification were reported. The complex was stable up to 90 min with a logP of -2.91. Maximum ligand binding (65%) was observed in the presence of 50 mg of HA; a major portion of the activity was excreted through the kidneys. By excluding the excretory organs, gastrointestinal tract organs, including the liver, intestine, and colon, showed significant uptake; however; the bone uptake was low after 30 min (<1%). The data were confirmed by sequential imaging at 30-90 min following the injection. The high solubility and anionic properties of the complex led to high renal excretion and low HA uptake; therefore, the complex failed to demonstrate bone imaging behaviors.
